# Culture-Negative Early-Onset Neonatal Sepsis — At the Crossroad Between Efficient Sepsis Care and Antimicrobial Stewardship

**DOI:** 10.3389/fped.2018.00285

**Published:** 2018-10-09

**Authors:** Claus Klingenberg, René F. Kornelisse, Giuseppe Buonocore, Rolf F. Maier, Martin Stocker

**Affiliations:** ^1^Pediatric Research Group, Faculty of Health Sciences, University of Tromsø-Arctic University of Norway, Tromsø, Norway; ^2^Department of Pediatrics and Adolescence Medicine, University Hospital of North Norway, Tromsø, Norway; ^3^Division of Neonatology, Department of Pediatrics, Erasmus MC-Sophia Children's Hospital, Erasmus University Medical Center, Rotterdam, Netherlands; ^4^Department of Molecular and Developmental Medicine, University of Siena, Siena, Italy; ^5^Children's Hospital, University Hospital, Philipps University of Marburg, Marburg, Germany; ^6^Neonatal and Pediatric Intensive Care Unit, Children's Hospital, Lucerne, Switzerland

**Keywords:** neonate, sepsis, blood culture, C-reactive protein, procalcitonin

## Abstract

Sepsis is a leading cause of mortality and morbidity in neonates. Presenting clinical symptoms are unspecific. Sensitivity and positive predictive value of biomarkers at onset of symptoms are suboptimal. Clinical suspicion therefore frequently leads to empirical antibiotic therapy in uninfected infants. The incidence of culture confirmed early-onset sepsis is rather low, around 0.4–0.8/1000 term infants in high-income countries. Six to 16 times more infants receive therapy for culture-negative sepsis in the absence of a positive blood culture. Thus, culture-negative sepsis contributes to high antibiotic consumption in neonatal units. Antibiotics may be life-saving for the few infants who are truly infected. However, overuse of broad-spectrum antibiotics increases colonization with antibiotic resistant bacteria. Antibiotic therapy also induces perturbations of the non-resilient early life microbiota with potentially long lasting negative impact on the individual‘s own health. Currently there is no uniform consensus definition for neonatal sepsis. This leads to variations in management. Two factors may reduce the number of culture-negative sepsis cases. First, obtaining adequate blood cultures (0.5–1 mL) at symptom onset is mandatory. Unless there is a strong clinical or biochemical indication to prolong antibiotics physician need to trust the culture results and to stop antibiotics for suspected sepsis within 36–48 h. Secondly, an international robust and pragmatic neonatal sepsis definition is urgently needed. Neonatal sepsis is a dynamic condition. Rigorous evaluation of clinical symptoms (“organ dysfunction”) over 36–48 h in combination with appropriately selected biomarkers (“dysregulated host response”) may be used to support or refute a sepsis diagnosis.

## Introduction

Neonatal sepsis is a clinical manifestation of a systemic infection during the first 28 days of life, usually classified as early-onset (<48–72 h) and late-onset sepsis (>48–72 h), depending on the age at onset of the sepsis episode (1). The inability to isolate a microbial pathogen does not exclude sepsis (2, 3). The 2016 consensus definition of sepsis in adults (Sepsis-3) states that sepsis is “a life-threatening condition caused by a dysregulated host response to infection and organ dysfunction” (3). There are also ongoing discussions regarding an adapted definition of pediatric sepsis beyond the neonatal period, similar to the Sepsis-3 definition (4, 5). However, there is unfortunately no uniform consensus definition for neonatal sepsis (2, 6, 7). Many neonates are therefore diagnosed with a “probable or possible” sepsis or a “presumed symptomatic infection but no bacterial cause identified” (8); conditions often referred to as “culture-negative sepsis”(9).

Most publications on neonatal sepsis from high-income countries only include culture-confirmed cases. The large number of neonates treated with antibiotics for a culture-negative sepsis are largely ignored in epidemiological studies. Thus, there is a paucity of data on the latter condition. However, neonates with culture-negative sepsis contribute to the high antibiotic consumption seen in neonatal units (10, 11). Moreover, there is a wide variation in antibiotic use between neonatal intensive care units (NICUs) and countries, with no difference in infection-attributable morbidity and mortality (10, 12). This indicates probable overuse of antibiotics in some NICUs, which again may have important adverse short- and long-term consequences for the infant's individual health (13–15). On the other hand, delayed antibiotic therapy in truly infected neonates may result in increased mortality and morbidity (16). Therefore, culture-negative neonatal sepsis is at the crossroad between efficient sepsis care and antimicrobial stewardship programs.

The main objective of this paper is to critically review the controversial term culture-negative neonatal sepsis (9, 17), with a focus on early-onset neonatal sepsis (EONS). We discuss how we can combine efficient sepsis care with antimicrobial stewardship during first days and weeks of life. Finally, we will discuss possible strategies for defining EONS in the absence of a positive blood culture.

## Efficient sepsis care—basic elements

### Epidemiology

In most high-income countries, the incidence of culture-confirmed EONS has decreased or remained relatively stable around 0.4–0.8 cases per 1000 live-born term infants over the last decade (10, 18–21). Although the incidence of EONS is substantially higher in preterm infants, the majority of patients with EONS are delivered at term (22). Robust epidemiological data on culture-negative sepsis are limited. A number of different studies published since 2012 report that infants receiving systemic antibiotic therapy for culture-negative sepsis outnumber infants receiving therapy for culture-confirmed sepsis by a factor of six to 16 (Table 1). The reason for the high number of culture-negative cases is not clear, and diagnostic criteria used in the different publications vary substantially (10, 11, 23, 24, 26, 27). Low levels of bacteremia or only small volumes of blood obtained from sick infants may be one explanation for the high number of culture-negative sepsis cases. Also maternal antibiotic treatment before or during delivery may theoretically in some cases mask detection of bacteremia in the newborn. However, one must also question whether over-diagnosis of sepsis among non-infected infants is an alternative explanation.

**Table 1:** Reported cases of culture-confirmed vs. culture-negative neonatal sepsis in selected studies published after 2012.

**Population and country**	**Ratio culture-confirmed vs. culture-negative sepsis cases**	**Definition culture-negative sepsis**
Term infants admitted to neonatal units in Norway over a 3 year period (*n* = 10 175) (10)	91: 1447 (1:16)	• Physician assigned ICD-10 diagnosis P36.9 • Clinical symptoms • Antibiotics ≥5 days
Term and preterm infants admitted to one neonatal unit in Norway and one in Denmark over a 3 year period (*n* = 2927) (23)	35: 203 (1:6)	• Physician assigned ICD-10 diagnosis P36.9 • Clinical symptoms • CRP > 10 mg/L • Antibiotics ≥3 days
Term and preterm infants admitted within first 24 h of life to a single neonatal unit in Austria (*n* = 851) (24)	31^x^: 209 (1:7)	• Clinical symptoms • Maternal risk factor or at least one abnormal laboratory marker (incl. CRP > 8 mg/L)
Term and preterm infants evaluated for sepsis in one neonatal unit in Canada (*n* = 1202) (25)	16: 107 (1:7)	• Born to mothers receiving intrapartum antibiotics. • Antibiotics started on the day of birth and continued for > 72 h despite negative culture.
Infants born after 34 weeks gestation with suspected EONS requiring antibiotics. A multi-center study in Europe and Canada. (*n* = 1710) (26)	27: 161 (1:6)	Based on a risk classification scheme including: • Maternal risk factors • Clinical symptoms • Laboratory findings (WBC < 5 x 10^9^/mL and/or CRP > 10 mg/L)
Infants born after 34 weeks gestation at a single institution in Switzerland over a 5-year period (*n* = 11 503) (27)	4: 48 (1:12)	• ≥2 clinical signs of sepsis within the first 7 days of life (temperature instability, irritability, or lethargy, feeding difficulties, capillary refill >2 s, apnea, tachycardia and/or tachypnea) • CRP > 20 mg/L • Antibiotics ≥7 days

x *11 cases of positive tracheal cultures indicating invasive infections were reported in the original paper, but excluded here*.

*WBC, white blood cells; CRP, C-reactive protein; ICD-10, International Classification of Diseases, 10th revision*.

Survivors of neonatal sepsis may suffer short- and long-term consequences (28, 29). Delayed recognition and therapy of sepsis increase the risk of morbidity and mortality and early antibiotic treatment of suspected sepsis is a corner stone of every sepsis care bundle (30). Therefore, clinicians have a low threshold to suspect neonatal EONS and to start empirical antibiotic therapy. Most probably due to a lack of a clear definition and a robust gold standard of the diagnosis neonatal sepsis, started therapy is often continued for 5–7 days, even in the absence of a positive blood culture, to minimize the risk of “partial treatment” of a potential true infection (11).

### Risk factors for EONS

Maternal group B streptococcal (GBS) colonization in the current pregnancy, GBS bacteriuria, a previous infant with invasive GBS disease, prolonged rupture of membranes (PROM; ≥18 h) and maternal fever (temperature ≥38°C; often interpreted as a sign of chorioamnionitis) are the risk factors most commonly associated with EONS. These risk factors are additive; the presence of more than one factor increases the likelihood of EONS (31, 32). EONS prevention strategies using a risk factor based approach suggest starting empiric antibiotics based on the presence of one or more risk factors. These strategies decrease the incidence of GBS-EONS (33, 34), but have poor predictive ability, leading to high number of mothers and infants being treated with antibiotics. A quantitative model assessing the indication for empiric therapy including degree of maternal fever, duration of PROM and degree of prematurity seem to perform better, and may reduce the number of infants receiving antibiotics (35).

Reports and guidelines differ in their recommendations on how to act on risk factor based strategies (36). The NICE-guidelines do not specify whether the use of intrapartum antibiotic prophylaxis alter the subsequent EONS risk assessment and infant evaluation (8). In contrast, an EONS sepsis calculator developed by Escobar and co-workers incorporates that intrapartum antibiotic given >2–4 h prior to delivery ameliorates the clinical recommendations regarding EONS risk, and subsequent infant management (37). A recent international survey regarding neonatal sepsis showed a broad variability in management of neonates with only risk factors, whereas a newborn with risk factors in combination with clinical signs were uniformly started on antibiotic therapy (36).

### Clinical signs of EONS

EONS generally presents itself with respiratory distress, apnea, lethargy or irritability, temperature instability, and feeding difficulties. These symptoms are unspecific as many non-infected neonates display similar symptoms. During the first days of life, there is a dynamic adaption of different organ systems to extra-uterine life. A single-point, clinical assessment to diagnose EONS therefore seems impossible. Some guidelines suggest that a respiratory rate > 50 or 60/min may be suggestive of an infection (Table 2). However, in healthy infants the 95th percentile for the respiratory rate is 65/min at 2 h of age (41). Moreover, non-infected early term infants (37–38 weeks gestation) born by cesarean section have high rates of transient tachypnea (42). Feeding difficulties are also notoriously difficult to assess during the first 1–2 days of life. A capillary refill time > 2 s is included as a sign of EONS in some sepsis criteria (27, 43), while observational studies indicate that the normal refill time in neonates is at least 3 s (44, 45). These examples clearly show the limitations with single-point assessments of clinical signs. In preterm infants, symptoms from respiratory distress syndrome or clinical instability may be impossible to differentiate from sepsis. However, a number of preterm infants are delivered by cesarean section, before rupture of membranes and with no maternal signs of infection. Among these there is a very low risk for EONS (46). Recognizing a “low-risk situation” may provide the opportunity to limit routine empiric antibiotics for preterm infants even when there is some degree of respiratory distress. Nevertheless, there is a broad consensus to start antibiotic therapy in newborns with clinical signs suggestive of possible EONS (36)—the major challenge is when to diagnose this as sepsis or to discard a sepsis diagnosis and stop therapy!

**Table 2:** Selected examples of suggested neonatal sepsis definition from experts or societies.

**Authors or society**	**Criteria**	**Comment**
International pediatric sepsis consensus conference: Definitions for sepsis and organ dysfunction in pediatrics−2005 (38)	**Systemic inflammatory response syndrome (SIRS) criteria**−**1st week of life** • Heart rate < 180/min or < 100/min • Respiratory rate > 50/min • WBC > 34 × 10^9^/mL • Core temperature of > 38.5°C or < 36°C Sepsis; SIRS in the presence of suspected or proven infection	Evidence of infection vaguely described
European Medicines Agency definition from 2010 (39)	**Criteria for inclusion in a neonatal sepsis clinical trial** Clinical sign (temperature instability, cardiovascular instability, skin symptoms, respiratory instability, gastrointestinal symptoms, non-specific symptoms) Laboratory signs: Low/high WBCs, I/T-ratio > 0.2, Platelet count < 100, CRP > 15 mg/L or PCT > 2 ng/mL, glucose intolerance and metabolic acidosis	Laboratory signs unspecific and cut-offs with poor predictive ability and not age adapted (PCT)
Wynn and Polin −2018 (2)	**Hypothetical neonatal sequential organ failure assessment (nSOFA) score** Including the following 6 items with scores from 0 to 3: Respiratory status, cardiovascular status, platelet counts, absolute neutrophil count, renal function, and CNS function	Inclusion of WBC indices with poor predictive values.
Hakonsson−2017 (40)	**Definition of a clinical, culture-negative GBS sepsis (in retrospect)** • Relevant symptoms and/or a CRP ≥ 25 mg/L • GBS in the maternal vaginal/rectal swabs or superficial infant cultures • The initiation of antibiotic therapy in the infant	Clinical signs not specified
Norwegian Neonatal Society (10)	**Definition of a culture-negative sepsis (in retrospect)** • Clinical symptoms • CRP > 30 mg/L • Other known clinical reasons for increased CRP excluded • Received antibiotics ≥ 5 days	Clinical signs not specified. Duration of antibiotics as a criteria not useful prospectively

*CRP, C-reactive protein; GBS, group B streptococci; CNS, central nervous system; WBC, white blood cells; PCT, procalcitonin, I/T ratio-immature to total neutrophil ratio*.

### Sensitivity and positive predictive value of inflammatory markers

In this paragraph we will focus on inflammatory markers that are commonly used and commercially available today, namely the complete blood cell (CBC) count, the C-reactive protein (CRP), procalcitonin (PCT), and to some extent interleukins (e.g., interleukin [IL]-6 and IL-8). It is not possible within the scope of this article to review all biomarkers used for diagnostics, and we refer to recent reviews on this topic (47). In the future, rapid bedside point-of care tests using e.g., microarray chips to quantify multiple cytokines and acute phase reactants and other “multi-omic” approaches may become excellent and improved diagnostic tools (47, 48).

The diagnostic value of the CBC-count and differential with regard to neonatal sepsis has been extensively evaluated (7, 49, 50). In general, CBC components are neither very sensitive nor specific for EONS (49, 50). The white blood cell (WBC) and the absolute neutrophil counts (ANC) are most informative when very low (49, 50). There is a very wide range between the lower and upper normal limits for WBC and ANC. Increased band form neutrophils, represented by high immature-to-total neutrophil ratio (IT-ratio), is often suggested to indicate sepsis. However, large intra- and inter-laboratory variation in interpretation limits its use (49, 51). Early onset thrombocytopenia is commonly associated with feto-maternal conditions and not very predictive for an infection (52). In the setting of an EONS evaluation, a low platelet count has a low sensitivity and a low positive predictive value (49, 53). Thus, CBC components used alone are inaccurate to define neonatal sepsis in the absence of a positive blood culture (7).

CRP is the most extensively studied acute-phase reactant in neonates. It is widely available and its determination is simple, fast, and cost-effective (54, 55). The mean CRP level in healthy term infants undergoes a physiological rise from ~1 mg/L at 12 h of age to ~4 mg/L at 48 h of life (55). Preterm infants have a less pronounced CRP response compared to term infants (56). A CRP cut-off value at 10 mg/L is often suggested as the upper normal limit (54). However, the 95th percentile of CRP in healthy infants at 48 h of age is 12–14 mg/L (55, 57, 58). Moreover, non-infected infants may have CRP values up to at least 40–50 mg/L, in particular after vaginal delivery (58, 59). Thus, a substantial number of healthy infants will have CRP values > 10 mg/L. CRP levels at the time of initial EONS-evaluation have a low sensitivity (25), because EONS usually originates during labor or at delivery and it then takes 12–24 h for CRP to rise (54). The positive predictive value of an elevated CRP value > 10 mg/L for possible or proven EONS has also consistently been low. This is mainly because there are multiple non-infectious conditions associated with an inflammatory reaction in the perinatal period, including a complicated labor and delivery, meconium aspiration, intraventricular hemorrhage, and tissue injury (25, 54, 60).

Over the last decade, PCT has emerged as a promising and increasingly used sepsis biomarker in neonates. PCT, like CRP, does not cross the placenta; hence is not affected by maternal fever in labor (61). PCT levels start to rise 2 h after start of a septic insult and peak by 12 h (62). The earlier rise after onset of the infectious stimulus makes PCT an attractive alternative to CRP (63). However, there has been few high-quality studies reporting data on the clinical usefulness of PCT at disease onset in “ruling in” EONS (63). Moreover, the interpretation of PCT values is challenging due to a strong physiological rise and fall during the first 72 h of life. Other perinatal factors, such as chorioamnionitis, hypoxemia, perinatal asphyxia, and maternal preeclampsia, can also cause PCT to increase (64). Dynamic reference values of PCT in neonates with and without EONS have been published (57, 65, 66).

Interleukins, predominantly IL-6 and IL-8, are used routinely in some countries and are considered as sensitive “early warning biomarkers” (47, 67, 68). The advantage of using interleukins is the early spike after an infectious insult that may add in early diagnosis. However, due to their short half-life one may not catch the peak level and this may limit sensitivity. Thus, when used they should always be combined with a “later marker” e.g., CRP (67).

In summary, the positive predictive values of the above-mentioned inflammatory markers are relatively low. The reason for this is mainly due to perinatal inflammatory reactions triggered by factors during delivery or in the early postnatal period. Still, many clinicians use abnormal inflammatory markers at disease onset as one reason to prolong antibiotic therapy in infants with a negative blood culture (36).

## Antimicrobial stewardship in the neonatal unit

### Prolonged antibiotic therapy in the neonatal period and associated risks

The duration of antimicrobial therapy in infants with negative blood cultures is controversial, and there is little evidence to inform practice (8, 31). In the SCOUT-study researchers assessed antibiotic consumption for different conditions among 2500 term and preterm infants (11). Culture-proven infection and necrotizing enterocolitis (NEC) accounted for only 6.9% of antibiotic use. In contrast, prolonged (≥ 5 days) antibiotic therapy for pneumonia or culture-negative sepsis accounted for 26% of total antibiotic use and therapy was continued for median (range) seven (5–14) days for these conditions. The UK NICE-guidelines states that the usual duration of antibiotic treatment for babies with a positive blood culture, and for those with a negative blood culture but in whom there has been strong suspicion of sepsis, should be 7 days (8). In observational studies, infants with culture-negative sepsis are commonly treated for 5–7 days and mortality is very low (10, 11, 23). However, due to variable sepsis definitions it is not possible to assess “safety” vs. a clear possibility of harm due to overtreatment (69). A recent study from India compared the efficacy of 7 vs. 10 days duration of intravenous antibiotics for culture-proven septic neonates and found no difference (70).

There is growing evidence that prolonged antibiotic therapy in preterm infants without proven infection increases the risk of mortality, bronchopulmonary dysplasia, NEC, retinopathy, and periventricular white matter damage (13, 71). The question if antibiotic therapy is a perpetrator or an innocent bystander is still not completely clear, but we must seriously consider that the “administration of antibiotics itself may contribute to mortality and morbidity among preterm infants” (72). It has also been known for decades that overuse of antibiotics paves the way for development of multi-resistant bacteria and there is an urgent call for rational use of antibiotics (73). Unfortunately, the cost of antibiotic resistance for the community compared to the potential risk of delayed or insufficient treated infection is a weak argument for the clinician at the bedside. With the growing knowledge of antibiotic use and the cost on the individual‘s own health via the collateral damage on their microbiota, this may change (74). Recent data indicate that the perturbations of the non-resilient microbiota in early life may influence both the immune system and stem cell populations with potential negative impact on future health (75–77). Therefore, pediatricians and neonatologists need to measure risk and benefits of every dose of antibiotics for culture-negative situations. The hospitals and lead clinicians in neonatal units have an independent responsibility to educate and keep staff updated about current knowledge of the harmful effects of unnecessary antibiotic use in the neonatal period (78). Recent publications reporting results from antimicrobial stewardship programs in NICUs highlight both the potential for improving antibiotic prescription practice, but also challenges when trying to reduce broad-spectrum antibiotic therapy in vulnerable very preterm infants (79, 80).

### Appropriate blood culture diagnostics

A positive microbial culture from a normally sterile site (blood) is frequently used as the gold standard to define neonatal sepsis (2). In the UK, around 50% of all blood cultures obtained in the neonatal period were taken on the day of birth, but only 0.8% of these were positive (81). Thus, the majority of blood cultures are negative in neonates who are evaluated with risk factors or clinical signs of EONS. What is the sensitivity of blood cultures in neonates? Schelonka analyzed, in a laboratory-based study, a range of blood volumes containing known concentrations of common neonatal pathogens injected into pediatric blood culture bottles. If organisms were present at very low densities [< 4 colony forming units (cfu)/mL] a blood culture volume of 1.0 mL was needed to detect bacteremia. Otherwise, blood culture volumes down to 0.5 mL were sufficient to detect most bacteremia's. In a similar *in-vitro* study, placental blood seeded with more than 10 cfu/mL of *E. coli* or GBS required only 0.25 mL blood to be consistently detected (82). Thus, blood cultures with a volume of 1.0 mL have excellent sensitivity even when the infant has low very levels of bacteremia, and blood culture volumes down to as little as 0.5 mL may be sufficient to detect moderate and high grade bacteremia (83). Some authorities recommend obtaining at least two cultures (aerobic and anaerobic) before commencing antibiotics, but there are no neonatal data to support this, and usual practice is to take only one aerobic blood culture bottle before starting antibiotic treatment (84).

Quantitative blood culture methodology (cfu/mL) is not routinely used. However, blood culture time to positivity (TTP) correlates to level of bacterial density as TTP is inversely proportional to the inoculated bacterial concentration. Using modern continuous monitoring blood culture system, median TTP of blood cultures in neonatal sepsis is 9–18 h for true pathogenic bacteria (85–89). For GBS and *E. coli* around 96–100% are positive by 36 h (85, 86, 89), whereas coagulase negative staphylococci may take up to 48 h to be detected. Maternal intrapartum antibiotic therapy does not seem to delay TTP (85, 90). Thus, considering TTP aids in clinical interpretation of blood culture results and is the reason why central guidelines suggest stopping antibiotics if culture results are negative at 36–48 h (8, 31). Ensuring a sufficient blood culture volume (minimum 0.5 mL) and adequate routines for 24/7 immediate entry of blood culture bottles into the blood culture system are essential elements in this diagnostic algorithm (84, 87, 91).

Modern molecular methods may potentially represent more rapid, sensitive, and specific ways of identifying bacteria. These methods include conventional and real-time polymerase chain reaction (PCR) assays targeted at universal and specific gene sites. A systematic review from 2017 concluded that molecular assays have the advantage of producing rapid results and may perform well as “add-on” tests, but cannot replace microbial cultures in the diagnosis of neonatal sepsis (92, 93). This may change in the future. A recent publication reports promising results using multiplex real-time PCR in late-onset neonatal sepsis with results potentially available within 4 h of blood sampling and good concordance with blood culture results (94).

### Negative predictive value of inflammatory markers

In contrast to the low positive predictive value, the negative predictive value of some inflammatory markers like CRP and PCT, and in combination with ILs, are good (26, 54, 67). Available evidence indicates that PCT may have the highest negative predictive value for severe, invasive bacterial infections in neonates (95, 96). Some high-quality intervention studies also show superiority of biomarker-guided duration of antibiotic therapy in neonatal (early- or late-onset) sepsis compared to a standard protocol (26, 67, 68, 97). The NICE-guideline states that if continuing antibiotics for longer than 36 h despite negative blood cultures, one should review the baby at least once every 24 h. Each day one should consider whether it is appropriate to stop antibiotic treatment, taking account of the level of initial clinical suspicion of infection, the baby's clinical progress and current condition, and whether there are “reassuring” levels and trends of CRP (8). One potential weakness with this high-quality guideline is the lack of discussion around which CRP values that can be interpreted as “reassuring.” After implementing the NICE guidelines recommending measuring CRP 18–24 h after commencing antibiotics, one report in fact showed that this led to more investigations and longer duration of antibiotic treatment (98).

A recent large randomized trial showed that PCT-guided decision-making was superior to standard care in reducing antibiotic therapy in neonates with suspected EONS. Clinicians may still “struggle” to become familiar with postnatal age specific PCT cut-off values over the first few 72 h of life which may confound the interpretation of a negative and a positive PCT value for the diagnosis of EONS. Therefore, a web-based guide for PCT-guided decision-making will hopefully be published in 2018-2019 (personal communication). A survey performed in 2011-2012 in Europe, North-America and Australia showed that PCT at that time was not as widely used as CRP to diagnose EONS (36). However, like for CRP, PCT seems to be an excellent additional tool to rule out EONS as long as age specific values are taken into account.

## Future definition of neonatal sepsis

The International Classification of Diseases, 10th Revision (ICD-10), defines different subtypes of bacterial sepsis in the newborn (P36). When specific pathogens are identified in the blood culture, the codes P36.0-8 are applied. For all other cases, e.g., when the clinician “believes” the newborn has sepsis the code P36.9 “unspecified bacterial sepsis” is applied. Regional differences in the use of P36.9 clearly exemplifies the need for a uniform sepsis definition (10). Both the US and the UK EONS-guidelines present the typical clinical symptoms that may indicate EONS, but do not specify clinical criteria defining sepsis in the absence of a positive blood culture (8, 31). Examples of some suggested neonatal sepsis criteria are presented in Table 2.

How can we define sepsis in neonates with a negative blood culture? Current adult sepsis definition is a “dysregulated host response to infection and organ dysfunction.” An adoption of this definition for neonatal sepsis may be possible, but the terms “dysregulated host response,” “infection,” and “organ dysfunction” need to be defined for the neonatal population. Interestingly, there are no risk factors included in the new adult Sepsis-3 definition, although risk factors also exist for sepsis in the adult population. Risk factors for EONS are well anchored in the sepsis framework for pediatricians and neonatologists. Clinical symptoms of sepsis may overlap with non-inflammatory conditions and we suggest that parameters reflecting a host inflammatory response (“dysregulated host response”) need to be included in order to establish a sepsis diagnosis in the absence of growth in a blood culture. Moreover, the biochemical picture of neonatal sepsis changes over the first days after antibiotics has been commenced for a suspected sepsis episode, and the kinetics of inflammatory biomarkers could also be included in a future sepsis definition. Finally, there is an urgent need for an international definition of organ dysfunction in neonates, which for the first days of life require a dynamic evaluation. Wynn and Polin recently called for a consensus definition of neonatal sepsis (5). As a group of European authors on neonatal sepsis we strongly agree with this call for action.

## Future strategies for efficient management of culture-negative sepsis

Two important factors may substantially reduce the number of cases coined as culture-negative sepsis.

First, available evidence indicate that blood cultures in neonates are highly sensitive, at least down to volumes of 0.5–1.0 mL. In neonatal units with available equipment and resources for obtaining blood cultures this diagnostic procedure must always be performed prior to commencing antibiotic therapy, with adequate aseptic technique and drawing a sufficient volume, preferably 1 mL and minimum 0.5 mL (9). We suggest to clearly record in the patient chart the blood culture volume taken. Automated 24-h electronic systems for reporting negative or positive (i.e., growth detected) blood culture results at 36–48 h incubation should be implemented in each hospital to support clinical decision-making of stopping antibiotics. Unless there is a strong clinical or biochemical indication to prolong antibiotics physician need to trust the culture results when an adequate volume is obtained and to stop antibiotics for suspected sepsis within 36–48 h. Our recommendations are in line with a recently published perspective article entitled “ending the culture of culture-negative sepsis in the neonatal ICU” where the authors urgently ask clinicians to trust negative cultures (9).

Second, a robust and pragmatic neonatal sepsis definition may reduce subjective variations in antibiotic use and support clinicians providing “appropriate” antibiotic therapy to those infants who need antibiotics. EONS is a dynamic and complex condition. A static definition of clinical symptoms reflecting organ dysfunction at a single point (disease onset) is most likely too unspecific to define EONS and guide antibiotic therapy. A combination of risk factors, symptoms at disease onset combined with development of symptoms upon evaluation after 36–48 h is an alternative approach. This would concomitantly be in line with clinical guidelines strongly suggesting reviewing the infant and deciding whether one should stop treatment of suspected EONS after 36–48 h. Escobar and co-workers developed an electronic sepsis calculator applicable for newborns ≥34 weeks' gestation including both quantitative maternal risk factors and clinical symptoms after birth. Implementation of this calculator in routine clinical care has been associated with a substantial and concomitantly and probably safe reduction in the number of infants commenced on antibiotics for suspected EONS in several countries (35, 99, 100). We believe a similar strategy for diagnosing EONS; including development of clinical symptoms over 36–48 h combined with a biomarker-guided approach may further reduce the unnecessary prolonged duration of antibiotic treatment. CRP and PCT, optionally combined with interleukin 6 or 8, are currently the most attractive biomarkers for this purpose.

In conclusion, there is a need to align the road toward efficient sepsis care with the road toward antimicrobial stewardship in the neonatal unit. Early antibiotic therapy for truly infected newborns is crucial for an optimal outcome. On the other hand, prolonged antibiotic therapy for non-infected newborns is harmful for their microbiota, for their future health and for the community.

## Author contributions

CK wrote the first draft of the manuscript. RK, GB, RM, and MS critically revised the manuscript for important intellectual content. All authors read and approved the final manuscript.

### Conflict of interest statement

The authors declare that the research was conducted in the absence of any commercial or financial relationships that could be construed as a potential conflict of interest.
